# Evaluation of the cell survival curve under radiation exposure based on the kinetics of lesions in relation to dose-delivery time

**DOI:** 10.1093/jrr/rru090

**Published:** 2014-10-29

**Authors:** Yusuke Matsuya, Kaori Tsutsumi, Kohei Sasaki, Hiroyuki Date

**Affiliations:** 1 Graduate School of Health Sciences, Hokkaido University, Kita-12, Nishi-5, Kita-ku, Sapporo 060-0812, Japan; 2 Faculty of Health Sciences, Hokkaido University, Kita-12, Nishi-5, Kita-ku, Sapporo 060-0812, Japan; 3 Faculty of Health Sciences, Hokkaido University of Science, Maeda 7-15, Teine-ku, Sapporo 006-8585, Japan

**Keywords:** microdosimetric–kinetic model, continuous irradiation, dose rate effects, linearity of high dose region

## Abstract

We have investigated the dose rate effects on cell damage caused by photon-beam irradiation. During a relatively long dose-delivery time with a low dose rate, lesions created in cells may undergo some reactions, such as DNA repair. In order to investigate these reactions quantitatively, we adopted the microdosimetric–kinetic (MK) model and deduced a cell surviving fraction (SF) formula for continuous irradiation. This model enabled us to estimate the SF from dose and dose rate. The parameters in the MK model were determined so as to generate the SF, and we attempted to evaluate the dose rate effects on the SF. To deduce the cell-specific parameters in the SF formula, including the dose rate, we performed a split-dose experiment and a single-dose experiment with a constant dose-delivery time (10 min) (to retain the condition for equivalent behavior of cell lesions) by means of a clonogenic assay. Then, using the MK model parameters, the SFs were reproduced for a variety of dose rates (1.0, 0.31, 0.18, 0.025 and 0.0031 Gy/min) and were compared with reported experimental data. The SF curves predicted by the MK model agreed well with the experimental data, suggesting that the dose rate effects appear in the kinetics of cell lesions during the dose-delivery time. From fitting the analysis of the model formula to the experimental data, it was shown that the MK model could illustrate the characteristics of log-SF in a rectilinear form at a high dose range with a relatively low dose rate.

## INTRODUCTION

Irradiation with photons such as X-rays and γ-rays is widely used in radiation therapy and diagnostics. Currently, the radiation weighting factor (*W*_R_) and the relative biological effectiveness (RBE) of photon beams are defined to be unity [1]. However, it is known that damage to the tissue or cells with photon irradiation depends on photon energy and dose rate [2]. We previously evaluated dependence on photon energy in relation to DNA damage number and RBE at a survival level of 37% [3]. Regarding dependence on the dose rate, although the effects have been well known, findings have rarely been reported, particularly concerning relatively long-term exposure to photon beams. Consequently, we sought to evaluate the biological effects in reference to both the radiation energy and the dose rate.

The effect on cells after radiation exposure is measured by the surviving fraction (SF), which can be determined by means of colony assay [4, 5]. For formulating the SF, the linear–quadratic (LQ) model has been applied to experimental data and used to conduct treatment planning for cancer in current radiotherapy [6, 7]. In the LQ model, the SF is given as a function of absorbed dose (*D*) in Gy with two coefficients, *α* and *β*, where *α* is the proportionality factor to *D* [Gy^−1^] and *β* is the proportionality factor to *D*^2^ [Gy^−2^]. These coefficients are determined empirically from clinical data or simply by fitting the LQ formula to the experimental cell data of SF. Although the LQ model is widely used, the model does not represent the dose rate effect explicitly, in which only one set of parameters (*α* and *β*) are determined as a function of dose (accumulated dose).

When aiming to deliver dose to an irradiation target (i.e. a tumor) at a prescribed level, we invariably need a time span of irradiation at a certain dose rate, which depends on the equipment in the facility. In an effort to attain tumor control, hypo-fractionated radiation therapy or real-time tumor-tracking radiation therapy (RTRT) is performed to treat cancer [8, 9]. Such treatment planning requires a large absorbed dose per fraction or a protraction of the dose-delivery time to 1–10 min or longer per fraction. During a long dose-delivery time, the repair processes for sublethal damage (SLD) may occur appreciably during the irradiation period [10, 11]. Therefore, determining cell behavior during dose-delivery is important for quantifying cell fate in response to radiation. Although a dose–time relationship based on the LQ formalism has been reported by many investigators [12–16], prediction using the relationship is yet to be verified [16]. Meanwhile, as a model that reflects the kinetics of cell lesions, the microdosimetric–kinetic model has been proposed by Hawkins in 1994 [17] (referred to hereafter as the MK model). The MK model was presented as a combination of the repair–misrepair (RMR) model of Tobias *et al*. [18] and the lethal–potentially lethal (LPL) model of Curtis *et al*. [19]. In 1996, the model was developed for continuous irradiation, which considers the kinetics of cell lesions during the dose-delivery time [20]. However, only a few investigations have been published reporting on continuous irradiation [21, 22]. There are some issues to be resolved, such as the dose rate effect on the cell survival curve during continuous irradiation, formulation of the survival curve accounting for the dose-rate effect, and determination of the specific parameters. Our interest was directed to the cell response under X-ray irradiation in order to quantify the endpoint of the bio-effects (cell-killing) in relation to the dose rate and radiation quality.

In this study, we deduce the SF formula in relation to the radiation energy and the dose rate based on the MK model, and investigate the relation between the dose rate effect and the shape of the SF curve. Cell-specific parameters in the MK model are determined, and by using these parameters the SF is predicted for a variety of dose rates to compare with experimental data reported by Metting *et al.* [23]. Finally, we show that the dose rate (or the dose-delivery time) has an influence on the fate of cell lesions, and the linear tendency of the log-survival curve in a high dose region is depicted.

### The microdosimetric–kinetic model

#### Theoretical base of the microdosimetric–kinetic model

In the MK model, the cell nucleus is divided into a few hundred domains (regarded simply as a pack of spheres), and it is supposed that some lesions, called potentially lethal lesions (PLLs), may arise in a domain after irradiation. The PLL is assumed to undergo one of four transformations: (i) it may be converted to an irreparable lethal lesion (LL) via a first-order process (rate constant for transformation is *a*); (ii) it may be converted to a LL via a second-order process (rate constant for transformation is *b_d_*); (iii) it may be repaired via a first-order process (rate constant for transformation is *c*); (iv) it may persist unchanged for a period of time *t_r_*, after which, if it is still present, it becomes a LL [20]. The PLLs are presumed to be DNA double-strand breaks (DSBs) in the MK model [3, 20]. Taking these pathways into account, rate equations for the number of lesions (PLLs, LLs) can be constructed according to the irradiation condition as described in the following subsections.

#### Single-instantaneous irradiation

The number of PLLs may arise as instant changes after a short-time (a single-instantaneous) irradiation. Using the rate constants (*a*, *b_d_* and *c*) for the transformations, a rate equation of the number of PLLs per domain is expressed as,

(1) $$\frac{dP}{dt}=-(a+c)P-2b_{d}P^{2}.$$

For the case of (*a + c*)*P* >> 2*b_d_P*^2^, Eq. (1) can be approximated by,

(2) $$\frac{dP}{dt}=-(a+c)P$$

and then we have

(3) $$P=k_{d}ze^{-(a+c)t}.$$

Here, *P* is the number of PLLs in a domain; *k_d_* is the average number of PLLs per domain per dose [Gy^−1^] just after the irradiation; *z* is specific energy deposited in the domain [Gy]; *t* is time after the irradiation [h] and satisfies 0 < *t < t_r_*. The rate equation of the LLs per domain (*L*) is expressed by

(4) $$\frac{dL}{dt}=aP+b_{d}P^{2}.$$

By solving Eq. (4) after the substitution of Eq. (3) into the terms in the right-hand side, we have

(5) $$L=Az+Bz^{2},$$

where

(6) $$A=\frac{a}{\left(a+c\right)}k_{d}+\frac{c}{\left(a+c\right)}k_{d}e^{-(a+c)t_{r}}\;\text{and}$$

(7) $$B=\frac{b_{d}k_{d}^{2}}{2(a+c)}[1-e^{-2(a+c)t_{r}}].$$

Assuming the Poisson distribution for the number of LLs in a cell nucleus, the average number of LLs per cell nucleus (*L*_n_) and the SF (*S*) for the single-instantaneous irradiation can be described as the expected value (with brackets) as follows:

(8) $$\begin{array}{cc} L_{n} & =N\left\langle L\right\rangle =N\left(A\left\langle z\right\rangle +B\left\langle z^{2}\right\rangle \right)\\ & =\left(\alpha_{0}+\gamma \beta_{0}\right)D+\beta_{0}D^{2}\\ & =\left(\alpha_{0}+\frac{y_{D}}{\rho \pi r_{d}^{2}}\beta_{0}\right)D+\beta_{0}D^{2}=-\ln S. \end{array}$$

Here,

(9) $$\alpha_{0}=NA,$$

(10) $$\beta_{0}=NB,$$

(11) $$k=Nk_{d},$$

(12) $$\gamma =\frac{y_{D}}{\rho \pi r_{d}^{2}}$$

and *N* is the number of domains; 〈*L*〉 is the average number of LLs per domain; *r_d_* and *ρ* represent the radius of the domain (0.5 μm) and the density of the domain (1.0 g/cm^3^), respectively; *D* is the absorbed dose (Gy); *k* is the number of PLLs per cell nucleus per Gy, which corresponds to the number of DSBs; *y_D_*-value is the dose mean lineal energy (keV/μm). Concerning the two parameters *α*_0_ and *β*_0_, we assumed that these are the specific parameters for single-instantaneous irradiation. In the single-instantaneous irradiation, the SF is given by Eq. (8), in which the exposure time of radiation is assumed to be short enough to neglect the dose-delivery time in this derivation.

#### Split-dose irradiation for determining cell-specific parameter (a + c)

In the MK model, (*a + c*) is a cell-specific value and can be determined by the split-dose irradiation with the following procedure. If we consider a domain exposed to two irradiations at different timings, *z*_1_ at *t =* 0 and *z*_2_ at *t = τ*, the numbers of PLLs per domain for *z*_1_ and *z*_2_ are given by:

(13) $$P_{1}=k_{d}z_{1}e^{-(a+c)t}\;\text{and}$$

(14) $$P_{2}=k_{d}z_{2}e^{-(a+c)(t-\tau )}.$$

The kinetics of PLLs per domain differs according to the magnitude relation between *t_r_* and *τ*. The rate equation for the LLs per domain (*L*) for *τ < t_r_* is expressed as:

(15) $$\frac{dL}{dt}=a(P_{1}+P_{2})+b_{d}(P_{1}+P_{2}{)}^{2}.$$

Then, we have,

(16) $$L=Az_{1}+Bz_{1}^{2}+Az_{2}+Bz_{2}^{2}+2B\left[\frac{1-e^{-2(a+c)(t_{r}-\tau )}}{1-e^{-2(a+c)t_{r}}}\right]e^{-(a+c)\tau }z_{1}z_{2}.$$

The average number of LLs for cell nucleus (*L*_n_) and the SF (*S*) is described by:

(17) $$\begin{array}{rl} L_{n} & =\alpha D_{1}+\beta_{0}D_{1}^{2}+\alpha D_{2}+\beta_{0}D_{2}^{2}\\ \;+2\beta_{0}\left[\frac{1-e^{-2(a+c)(t_{r}-\tau )}}{1-e^{-2(a+c)t_{r}}}\right]e^{-(a+c)\tau }D_{1}D_{2}\\ & =-\ln S. \end{array}$$

Here, *D*_1_ represents the dose absorbed by the population of cells at *t =* 0; *D*_2_ represents the dose absorbed by the cells at *t = τ*.

In the same manner, the rate equation for the LLs per domain (*L*), the numbers of LLs per domain (*L*) and per cell nucleus (*L*_n_), and SF (*S*) for *t_r_ ≤ τ* are given by:

(18) $$\frac{dL}{dt}=aP_{1}+b_{d}P_{1}^{2}+aP_{2}+b_{d}P_{2}^{2},$$

(19) $$L=Az_{1}+Bz_{1}^{2}+Az_{2}+Bz_{2}^{2}\;\text{and}$$

(20) $$L_{n}=\alpha D_{1}+\beta D_{1}^{2}+\alpha D_{2}+\beta D_{2}^{2}=-\ln S.$$

Substituting *τ* = 0 into Eq. (17) and taking the limit (*τ* → ∞) in Eq. (20), we have

(21) $$\ln S\left(0\right)=-\alpha D_{1}-\beta_{0}D_{1}^{2}-\alpha D_{2}-\beta_{0}D_{2}^{2}-2\beta_{0}D_{1}D_{2}$$

and

(22) $$\ln S(\infty )=-\alpha D_{1}-\beta_{0}D_{1}^{2}-\alpha D_{2}-\beta_{0}D_{2}^{2}.$$

Then, subtracting Eq. (21) from Eq. (22) gives

(23) $$\ln S(\infty )-\ln S\left(0\right)=2\beta_{0}D_{1}D_{2}.$$

On the other hand, the derivative of Eq. (17) is

(24) $$\frac{\text{1}}{S}\frac{dS}{d\tau }=\frac{-2\beta_{0}D_{1}D_{2}}{1-e^{-2(a+c)t_{r}}}\times \frac{d}{d\tau }[e^{-(a+c)\tau }-e^{-(a+c)(2t_{r}-\tau )}].$$

Finally, by taking the limit of *dS/dτ* as *τ* tends to zero, we have:

(25) $$\underset{\tau \to 0}{\lim}\frac{1}{S}\frac{dS}{d\tau }=2\beta_{0}D_{1}D_{2}(a+c)\left[\frac{1+e^{-2(a+c)t_{r}}}{1-e^{-2(a+c)t_{r}}}\right].$$

If we assume that (*a + c*)*t_r_* is greater than 3 as reported by Hawkins [20] and substitute Eq. (23) into Eq. (25), the value of (*a + c*) can be determined by the next equation:

(26) $$(a+c)=\frac{\underset{\tau \to 0}{\lim}\frac{\text{1}}{S}\frac{dS}{d\tau }}{\text{ln}\frac{S(\infty )}{S(0)}}.$$

#### Single-continuous irradiation

**Fig. 1.**
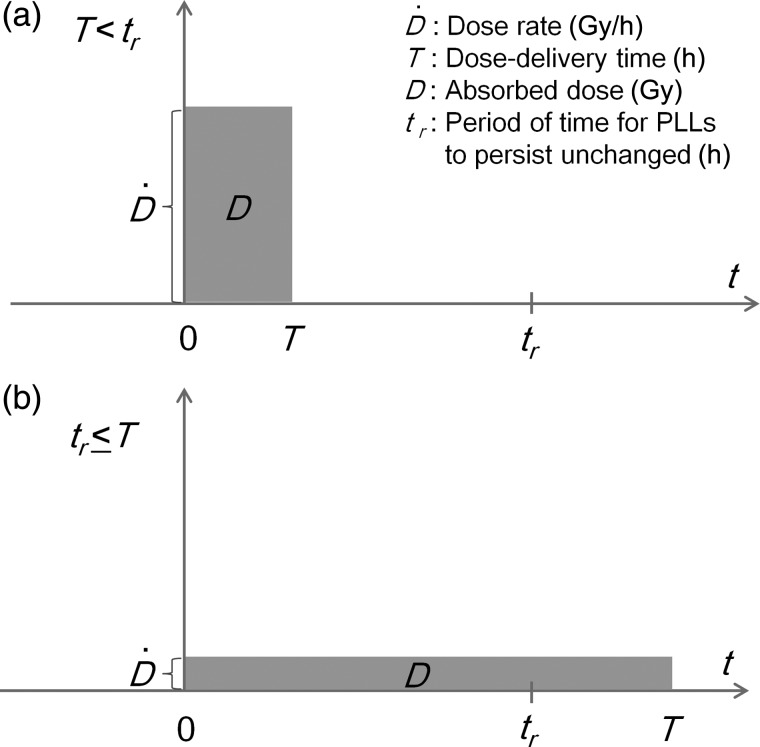
Two cases of single-continuous irradiation: (**a**) *T* < *t_r_* and (**b**) *t_r_* ≤ *T*. PLLs = potentially lethal lesions.

When we try to irradiate cultured cells (or bio tissues) to a prescribed dose level, it takes a certain amount of time (the dose-delivery time) in general. Here, if we consider a population of cells irradiated at a constant rate of $\dot{D}$ (in Gy per h) from *t* = 0 to *t* = *T*, the rate equation for the number of PLLs per domain should be considered for two cases, *T* < *t_r_* and *t_r_* ≤ *T* as in Fig. 1.

First, in the case of *T* < *t_r_* in Fig. 1a, a set of conditional equations are given assuming the constant rate of $\dot{z}=\dot{D}/N=z/T$ per domain as:

(27) $$\frac{dP_{d(1)}}{dt}=k_{d}\dot{z}-(a+c)P_{d(1)}\;(0<t\leq T)$$

(28) $$\frac{dP_{d(2)}}{dt}=-(a+c)P_{d(2)}(T<t\leq t_{r})$$

(29) $$\begin{array}{c} \frac{dP_{d(3)}}{dt}=-k_{d}\dot{z}e^{-(a+c)t_{r}}-(a+c)P_{d(3)}.(t_{r}<t\leq T+t_{r}) \end{array}$$

Then, the numbers of PLLs per domain for three time intervals, *P*_d(1)_, *P*_d(2)_, *P*_d(3)_, are given by

(30) $$P_{d(1)}=\frac{k_{d}\dot{z}}{a+c}[1-e^{-(a+c)t}]\;(0<t\leq T)$$

(31) $$\begin{array}{c} P_{d(2)}=\frac{k_{d}\dot{z}}{a+c}[1-e^{-(a+c)T}]\cdot e^{-(a+c)(t-T)}\;(T<t\leq t_{r}) \end{array}$$

(32) $$P_{d(3)}=\frac{k_{d}\dot{z}}{a+c}[-e^{-(a+c)t_{r}}+e^{-(a+c)(t-T)}]\;.\;(t_{r}<t\leq T+t_{r}).$$

From these solutions, the number of LLs per domain (*L*) for *T < t_r_* is expressed by

(33) $$\begin{array}{cc} L=\; & A\;\dot{z}\;T+\frac{2B(\dot{z}T{)}^{2}}{(a+c{)}^{2}T^{2}}\cdot \frac{1}{1-e^{-2(a+c)t_{r}}}\times [(a+c)T\\ & +e^{-(a+c)T}-1-e^{-(a+c)(2t_{r}-T)}+e^{-2(a+c)t_{r}}\\ & +(a+c)Te^{-2(a+c)t_{r}}]. \end{array}$$

The average number of LLs per cell nucleus (*L*_n_) and the SF (*S*) for *T < t_r_* can be described by using the expected value (with brackets) under the assumption that the number of LLs in a cell nucleus follows the Poisson distribution as

(34) $$\begin{array}{cc} & L_{n}=N\left\langle L\right\rangle \\ & \;=-\alpha_{0}\left\langle z\right\rangle -\frac{2\beta_{0}\left\langle z^{2}\right\rangle }{{\left(a+c\right)}^{2}T^{2}}\cdot \frac{1}{1-e^{-2\left(a+c\right)t_{r}}}\\ & \;\times [\left(a+c\right)T+e^{-\left(a+c\right)T}-1-e^{-\left(a+c\right)(2t_{r}-T)}\\ & \;\;+e^{-2\left(a+c\right)t_{r}}+\left(a+c\right)Te^{-2\left(a+c\right)t_{r}}]. \end{array}$$

If we assume that (*a + c*)*t_r_* is greater than 3 [20], Eq. (34) can be approximated by

(35) $$\begin{array}{cc} & L_{n}=-\alpha_{0}D-\frac{2\beta_{0}\left(D^{2}+\frac{y_{D}}{\rho \pi r_{d}^{2}}D\right)}{(a+c{)}^{2}T^{2}}\times [(a+c)T+e^{-(a+c)T}-1]\\ & -\ln S. \end{array}$$

Next, in the case of *t_r_* ≤ *T* in Fig. 1b, the rate equations are solved in the manner same as the case for *t_r_* ≤ *T* as:

(36) $$\frac{dP_{d(1)}}{dt}=k_{d}\dot{z}-(a+c)P_{d(1)}\;(0<t\leq t_{r})$$

(37) $$\frac{dP_{d(2)}}{dt}=k_{d}\dot{z}-(a+c)P_{d(2)}-k_{d}\dot{z}e^{-(a+c)t_{r}}\;(t_{r}<t\leq T)$$

(38) $$\frac{dP_{d(3)}}{dt}=-k_{d}\dot{z}e^{-(a+c)t_{r}}-(a+c)P_{d(3)}.(T<t\leq T+t_{r})$$

(39) $$P_{d(1)}=\frac{k_{d}\dot{z}}{a+c}[1-e^{-(a+c)t}]\;(0<t\leq t_{r})$$

(40) $$P_{d(2)}=\frac{k_{d}\dot{z}}{a+c}[1-e^{-(a+c)t_{r}}]\;(t_{r}<t\leq T)$$

(41) $$P_{d(3)}=\frac{k_{d}\dot{z}}{a+c}[-e^{-(a+c)t_{r}}+e^{-(a+c)(t-T)}].(T<t\leq T+t_{r})$$

Using Eqs (39)–(41), the number of LLs per domain (*L*) for *t_r_* ≤ *T* is expressed by

(42) $$\begin{array}{cc} & L=A\dot{z}T+\frac{2B(\dot{z}T{)}^{2}}{(a+c{)}^{2}T^{2}}\cdot \frac{1}{1-e^{-2(a+c)t_{r}}}\\ & \times \{\left(a+c)T[1-e^{-(a+c)t_{r}}{]}^{2}-1-e^{-2(a+c)t_{r}}+2e^{-(a+c)t_{r}}[(a+c)t_{r}+e^{-(a+c)t_{r}}\right]\}. \end{array}$$

Then, the average number of LLs per cell nucleus (*L*_n_) and the SF (*S*) is described by

(43) $$\begin{array}{cc} L_{n}=\; & N\left\langle L\right\rangle \\ = & -\alpha_{0}\left\langle z\right\rangle -\frac{2\beta_{0}\left\langle z^{2}\right\rangle }{(a+c{)}^{2}T^{2}}\cdot \frac{1}{1-e^{-2(a+c)t_{r}}}\\ & \times \{(a+c)T[1-e^{-(a+c)t_{r}}{]}^{2}-1-e^{-2(a+c)t_{r}}\\ & \;+2e{}^{-(a+c)t_{r}}[(a+c)t_{r}+e^{-(a+c)t_{r}}]\}. \end{array}$$

This equation can be approximated by

(44) $$\begin{array}{cc} & L_{n}=-\alpha_{0}D-\frac{2\beta \left(D^{2}+\frac{y_{D}}{\rho \pi r_{d}^{2}}D\right)}{(a+c{)}^{2}T^{2}}\{(a+c)T-1\}\\ & \;-\ln S. \end{array}$$

Finally, the descriptions of SF for *T < t_r_* and *t_r_ ≤ T* (Eq. (35) and Eq. (44)) are summarized as follows:

(45) $$\begin{array}{cc} -\ln S & =\left(\alpha_{0}+\frac{y_{D}}{\rho \pi r_{d}^{2}}F\beta_{0}\right)D+F\beta_{0}D^{2}\\ & =\alpha D+\beta D^{2}, \end{array}$$

where

(46) $$F=\left\{\begin{array}{c} \;f=\frac{2}{(a+c{)}^{2}T^{2}}[(a+c)T+e^{-(a+c)T}-1]\;(T<t_{r})\\ f^{\prime }=\frac{2}{(a+c{)}^{2}T^{2}}\left[(a+c)T-1\right]\;(t_{r}\leq T) \end{array}\right.$$

(47) $$D=\dot{D}T,$$

(48) $$\alpha =\alpha_{0}+\frac{y_{D}}{\rho \pi r_{d}^{2}}F\beta_{0},$$

(49) $$\beta =F\beta_{0}.$$

In Eq. (45), *α* includes *β*_0_, *y_D_* and *F*, while *β* includes *β*_0_ and *F*. According to Brenner *et al*. [16], *F* might correspond to the Lea-Catcheside time-factor *G*. Therefore, Eq. (45) provides the general form of SF for continuous irradiation conditions. Here, *α* and *β* are not constant but ought to decrease as the dose-delivery time is protracted. It should be noted that the decrease in *α* depends on the radiation quality, *y_D_* [keV/μm].

## MATERIALS AND METHODS

### Cell irradiation experiments

To deduce the cell-specific parameters (*a + c*), *α*_0_, *β*_0_ and *t_r_*, we performed a clonogenic assay and immunofluorescent staining with γ-H2AX antibody. We targeted Chinese hamster ovary cell lines, CHO-K1, taken from RIKEN Bio Resource Center in Japan (RCB0285). CHO-K1 cells were maintained in Dulbecco's modified Eagle's medium (DMEM, Sigma, St Louis, MO, USA) supplemented with 10% fetal bovine serum (FBS, Nichirei Biosciences Inc., Tokyo, Japan) at 37°C in a humidified 95% air and 5% CO_2_. The cells were cultured on tissue culture dishes with *ϕ* 60-mm or in *ϕ* 18-mm glass cover slips coated with type I collagen in tissue culture dishes with *ϕ* 35 mm. During the radiation exposure, cells were in a contact plateau state, which is an equivalent condition to that reported by Metting *et al*. [23].

**Fig. 2.**
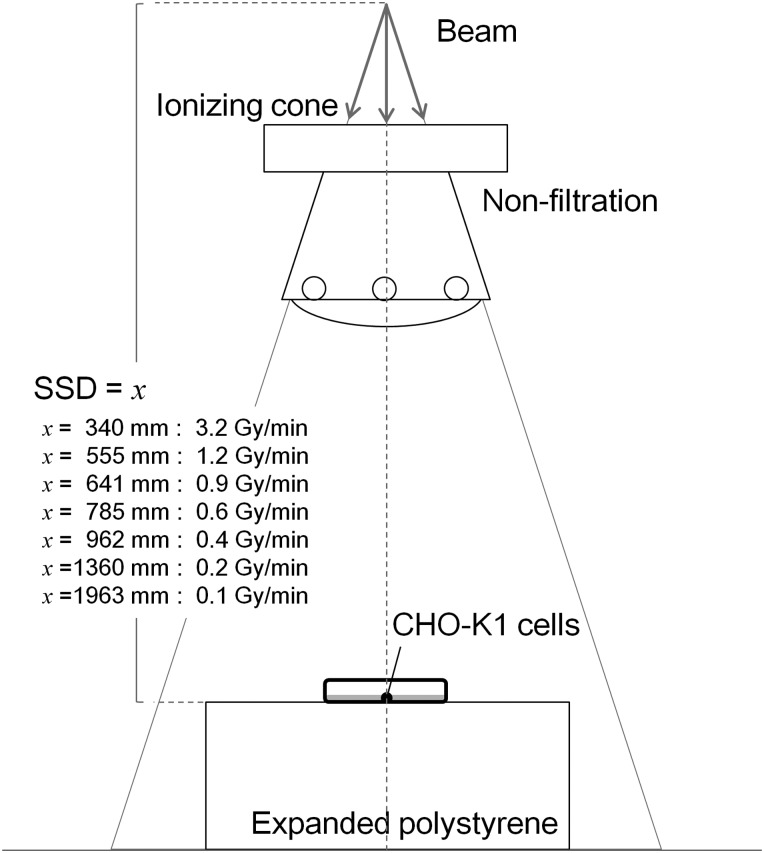
Schematic of the irradiation geometry for 250-kVp X-rays. The dose rates in water were measured using ionization chamber NE2751. We confirmed that these dose rates followed the inverse square law of the distance.

The cultured cells were irradiated with 250-kVp X-rays (STABILIPAN, Siemens, Concord, CA). We measured the dose rate in air at the surface of cell culture using a Farmer-type ionization chamber (model NE2571, Nuclear Enterprises Ltd) and determined the dose rate and absorbed dose for water according to TRS 277 (in air method) [24]. The dose rates in water were adopted as 0.1, 0.2, 0.4, 0.6, 0.9, 1.2 and 3.2 Gy/min by taking the inverse square law of the distance into account (Fig. 2).

In this study, two types of irradiation method, a split-dose irradiation and a single-dose irradiation, were employed. On one hand, to determine the MK parameter (*a + c*), we took the split-dose irradiation with an equal dose condition as *D*_1_ = *D*_2_ = 3.0 Gy (with 5 min dose-delivery time) for the interval (*τ*) from 0 to 4 h. On the other hand, to determine the MK parameters *α*_0_ and *β*_0_ for a variety of dose rates, the single-dose irradiation was conducted for a fixed irradiation time (10 min dose-delivery time) considering the same period for the progression of PLLs.

### Clonogenic assay

After the radiation exposure, cells were trypsinized and plated in tissue culture dishes (six-well plates). Next, the cells were cultured in a CO_2_ incubator for 14 days, replacing the DMEM every 2 days. Then, the cells were fixed with methanol and stained with 2% Giemsa solution (Kanto Chemical Co. Inc., Tokyo, Japan) to count the number of colonies per dish. Finally, the survival rate was determined from the colony counts with the plating efficiency of the non-irradiated cells.

### Immuofluorescent staining with γ-H2AX

The cells were exposed to 250-kVp X-rays of 1.0 Gy at 3.2 Gy/min. The cells were maintained for 30 min at 37°C in a humidified 95% air, 5% CO_2_ environment and fixed in an ice cold 4% paraformaldehyde solution with PBS for 10 min at room temperature. The fixed cells were rinsed three times with phosphate buffered saline (PBS). Then, the cells were permeabilized in ice-cold 0.2% Triton X-100 in PBS for 5 min, and blocked with a solution of 1% BSA-containing PBS for 30 min. After that, a primary antibody, γ-H2AX (abcam), diluted with a solution of 1% BSA-containing PBS (1:400) was fed on glass cover slips, and kept overnight at 4°C. The next day, the primary antibody was removed and rinsed three times with PBS, and secondary antibody, AlexaFluor594-conjugated goat-anti-rabbit (Molecular Probes, Invitrogen, Japan) diluted with a solution of 1% BSA-containing PBS (1:250), put onto cover slips and left for 2 h, then rinsed once with 1 μg/ml DAPI (4′,6-diamidino-2-phenylindolephenylindole)-containing methanol. The cells on the cover slips were stained with 1 μg/ml DAPI-containing methanol for 15 min in the incubator, and rinsed once with methanol.

The γ-H2AX foci detected in cell nuclei were observed using a High Standard all-in-one fluorescent microscope (BZ-9000; Keyence, Osaka, Japan) with Z-stack function. The images of the foci were reconstructed using the quick full focus (with 3D) in the cell nucleus. The number of the γ-H2AX foci was counted using Image J software to an accuracy at the visual level. This analysis was performed for a number of cells (>80 cells). We assumed that the number of DSBs is equivalent to the *k* value in the MK model [3], and the persistence time (*t_r_*) of the PLLs was deduced from this assumption.

### Determination of MK parameters, (*a + c*), *α_0_*, *β_0_* and *t_r_*

Assuming that *F* in Eq. (46) is constant for the dose-delivery time fixed at 10 min, the parameters (*α* and *β*) in Eq. (45) are fixed as constant values. Based on this assumption, the parameters in the MK model can be determined as follows:

1. The constant rate (DNA repair function), (*a + c*), is calculated from Eq. (26) with the split-dose experiment data.
2. *F* for 10 min is calculated from the (*a + c*) value in Eq. (46).
3. *α_0_* and *β_0_* are calculated using the parameters (*F*, *α* and *β*) that depend on *T*, *y_D_*, *r_d_* (0.5 μm) and *ρ* (1.0 g/cm^3^) according to Eqs (48) and (49), where the *y_D_*-value was estimated by Particle and Heavy Ion Transport code System (PHITS) [25–27] simulation and from references [28–31], *α* and *β* were determined by fitting the LQ formula to the experimental cell survival data (for single-dose irradiation for 10 min).
4. *t_r_* was determined by *α*, (*a + c*) and *k* (γ-H2AX foci number) following the reported equation [20, 21] i.e.
  (50) $$t_{r}=-\frac{\ln\frac{\alpha }{k}}{a+c}.$$

### Comparison of the MK formula with experimental data

By the use of the parameters (*a + c*), *α*_0_ and *β*_0_ determined so far, we predicted the cell survival curves for various dose rates, 1.0, 0.31, 0.18, 0.025 and 0.0031 Gy/min, and compared them with the experimental survival data reported by Metting *et al*. [23].

### Statistics

Statistical analyses were performed for the results of the γ-H2AX foci number and the conformance of the MK model to the experimental data obtained by Metting *et al*. [23]. For the former, the significant difference between the foci number in the control cell nucleus and that in the irradiated one was checked by an analysis of variance (ANOVA) test. For the latter, the degree of the reproduction was evaluated by the coefficient of determination (*R*^2^-value).

## RESULTS AND DISCUSSION

### Determination of the MK parameters

**Fig. 3.**
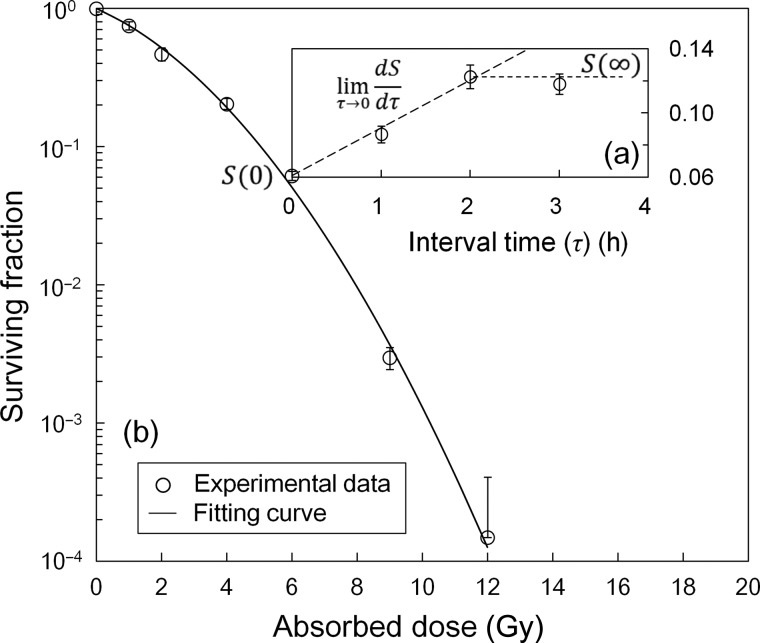
Surviving fraction obtained to deduce the MK parameters: (**a**) the result using a split-dose experiment with an equal dose condition: *D*_1_ = *D*_2_ = 3.0 Gy (5 min dose delivery time) for the interval (*τ*) from 0 to 4 h to determine (*a* + *c*) according to Eq. (26), (**b**) the result using a single-dose experiment in the case of 10 min (our) dose-delivery time to determine *α*_0_ and *β*_0_ using the value of (*a* + *c*) and Eqs (48) and (49). In Fig. 3a, *S*(0) and *S*(∞) represent the surviving fraction with an interval time (*τ*) with a split-dose exposure equalling zero and a limit of *τ* equalling infinity, respectively. The limit of *dS/dτ* was calculated as the gradient of three experimental points via spline interpolation.

**Fig. 4.**
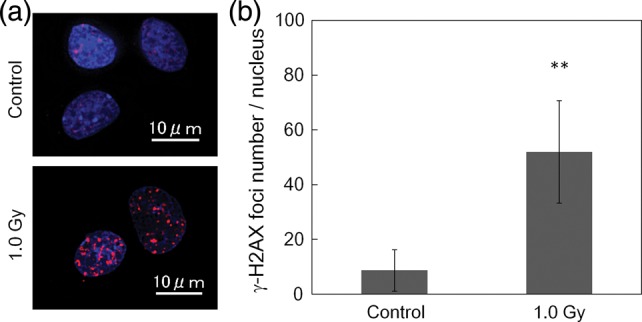
Results of γ-H2AX foci assay: (**a**) for foci observed in control cells and irradiated (1.0 Gy) cells, (**b**) for the number of foci. Each red point represents a γ-H2AX focus that is assumed to be a DNA double-strand break, and the blue area highlights the cell nucleus stained with DAPI. In Fig. 4b, the double asterisk signs indicate significant difference between foci number in the control cell nucleus and that in the irradiated one using the analysis of variance (ANOVA) test (*P =* 4.244 × 10^−43^).

Figure 3 shows the results of SF for the CHO-K1 cells: (a) for a split-dose experiment and (b) for the single-dose experiment with 10 min dose-delivery time. In Fig. 3b, the LQ formula was properly fitted to the survival data (*R*^2^ = 0.997). Figure 4a shows the image of γ-H2AX foci: each red point represents a DNA double-strand break and the blue area highlights a cell nucleus stained with DAPI. The number of DSBs per cell nucleus is given by Fig. 4b. The number of DSBs as the *k*-value for 1.0 Gy irradiation was found to be 52.0 ± 18.7 per nucleus. The MK parameters, (*a + c*), *α*_0_, *β*_0_ and *t_r_*, are listed in Table 1. The standard deviation (sd) of (*a + c*), *α*_0_, *β*_0_ and *t_r_* values in Table 1 was estimated by the formula of error propagation. The error (or rather ‘uncertainty’) of each parameter originates: for (*a + c*) from *S*(0), *S*(∞) and the initial slope of the split-dose recovery curve, for *β*_0_ from *β* and *F* associated with the error of (*a + c*), for *α*_0_ from the errors of *α* and *β*, and for *t_r_* from the errors of *α*, *k* and (*a + c*). The percentages of the standard deviation to the average for (*a+c*), *α*_0_, *β*_0_ and *t_r_*. were 16.8%, 16.3%, 8.72% and 18.2%, respectively.

**Table 1.** Parameters in the MK model determined in this study

*a + c* (h^−1^)	*α* (Gy^−1^)	*β*_0_ (Gy^−2^)	*t_r_* (h)	*γ* (Gy)	*y_D_* (keV/μm)	*r_d_* [μm]	*ρ* [g/cm^3^]
0.703 ± 0.118	0.208 ± 0.034	0.0436 ± 0.00380	7.62 ± 1.39	0.923	4.53	0.500	1.00

### Dose rate effects predicted by the MK model

**Fig. 5.**
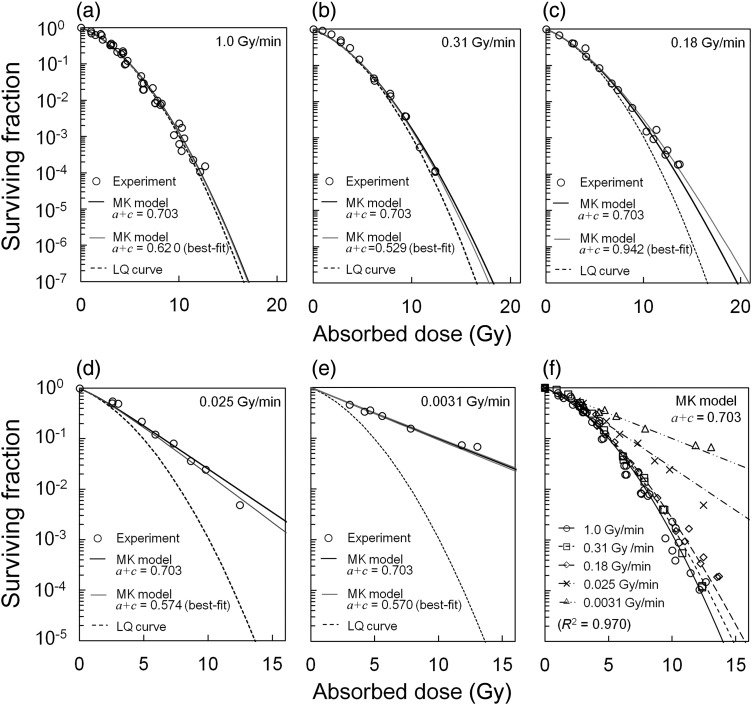
Cell survival curves and experimental data reported by Metting *et al*. [23]: (**a**)–(**e**) for the cell survival curves predicted by the MK model for various dose rates in comparison with the LQ curve according to Eq. (8) for an extremely high dose rate, (**f**) for the curves predicted by the MK model with experimental data for a dose up to ∼15 Gy.

The cell survival curves predicted using the MK parameters in Table 1 are derived for various dose rates, 1.0, 0.31, 0.18, 0.025 and 0.0031 Gy/min, as the solid black lines in Fig. 5a–e. As we can see in Fig. 5f, the MK formulae (Eqs (45) and (46)) for continuous irradiation accurately describe the SF that depends on the dose rate. More essentially, the SF can be expressed even for different dose rates by adopting appropriate values for the three parameters, (*a + c*), *α*_0_ and *β*_0_.

In Fig. 5d (0.025 Gy/min), we can recognize a significant difference between the SF estimated by the MK model with (*a* + *c* = 0.703) and the experimental point at 12.5 Gy, while the best-fit of the MK curve with (*a* + *c* = 0.574) gives a better fit. The best-fit value of (*a + c*) for each dose rate is shown in the legend of Fig. 5a–e, and the curve in solid gray line is appended in each figure. The uncertainty of the (*a + c*) value was estimated to be 0.703 ± 0.118 h^−1^, as in Table 1, suggesting that the MK model can express the dose rate effects with this value to a certain degree of accuracy. In contrast to the tendency of the dose rate effect here, the inverse dose rate effect (hypersensitivity) has been reported by some investigations [2, 32, 33]. To this, we cannot rule out the possibility that the inverse dose rate effects are related to the (*a + c*) value, and Fig. 5d represents this phenomenon by a low value of (*a + c*).

Recently, the effects of low dose-rate exposure on bio-cells have drawn keen interest. Besides the low dose-rate condition, the MK formula for continuous irradiation Eq. (45) must be of use for predicting damaging effects on normal tissues and tumors in radiotherapy.

### Cell survival curve at high dose range

Figure 5a–e also specifies the shapes for the cell survival curve at a higher dose range. At a high dose, the SFs by the MK model show a general tendency to be higher than those by the LQ curve. Here, the LQ curve was determined according to Eq. (8) for an extremely high dose rate. The curve for 1.0 Gy/min by the MK model formula is in good agreement with the LQ curve as shown in Fig. 5a. In contrast, as in Fig. 5b–e, the curve generated by the MK model deviates from the LQ curve at the higher dose region and displays a rectilinear shape that is more potent with decreasing dose rate.

It is generally known that the LQ model is useful over a wide dose range [34, 35], and it has been reported by Guckenberger *et al*. (2013) that accurate modeling of local tumor control in fractionated stereotactic body radiotherapy (SBRT) for a Stage I non-small-cell lung cancer (NSCLC) agrees well with the traditional LQ formalism from the viewpoint of the maximum likelihood tests and from the Bayesian reasoning using Bayes factors (BFs) [36]. In this regard, the LQ curves represent the special case for irradiation with a very high dose rate, as in radiotherapy.

The cell survival curve has been formulated by many models, e.g. the linear–quadratic–linear (LQ–L) model [37], universal survival curve (USC) [38], and non-lethal probability (NLP) model [39]. The linearity of the cell survival curve in the high dose region can be explained by Eq. (45), which employs the appropriate *β* parameter depending on the dose-delivery time. In fact, the quadratic factor *β* (*=Fβ*_0_) in the MK model expressed by Eq. (49) becomes smaller and smaller by protracting the dose-delivery time (*T*) to enhance the linearity of cell-survival curve at a higher dose range. Here, by taking the derivative of Eq. (45) and the limit (*D → ∞*), the natural log of the SF at a very high dose range is given as follows:

(51) $$\begin{array}{rl} -\ln S & =\left\{\alpha_{0}+\frac{2\gamma \beta_{0}{\dot{D}}^{2}}{(a+c{)}^{2}D^{2}}\left[(a+c)\frac{D}{\dot{D}}-1\right]\right\}D\\ \;-\frac{2\beta_{0}{\dot{D}}^{2}}{(a+c{)}^{2}D^{2}}\left[(a+c)\frac{D}{\dot{D}}-1\right]D^{2}\\ \frac{d}{dD}(-\ln S) & =\alpha_{0}+\frac{2\gamma \beta_{0}{\dot{D}}^{2}}{(a+c{)}^{2}D^{2}}+\frac{2\beta_{0}\dot{D}}{\left(a+c\right)}\\ & =\alpha_{0}+\frac{2\beta_{0}\dot{D}}{\left(a+c\right)}\left[1+\frac{\gamma \dot{D}}{(a+c)D^{2}}\right]\\ & \;\underset{D\to \infty }{\lim}\left[\frac{d}{dD}(-\ln S)\right]=\alpha_{0}+\frac{2\beta_{0}\dot{D}}{(a+c).} \end{array}$$

As is shown in Eq. (51), the natural log of the SF in the limit (*D → ∞*) depends on the dose rate $\left(\dot{D}\right)$ and the cell specific parameters ((*a + c*), *α*_0_ and *β*_0_), which gives the basis for the linearity of the survival curve at a very high dose.

**Fig. 6.**
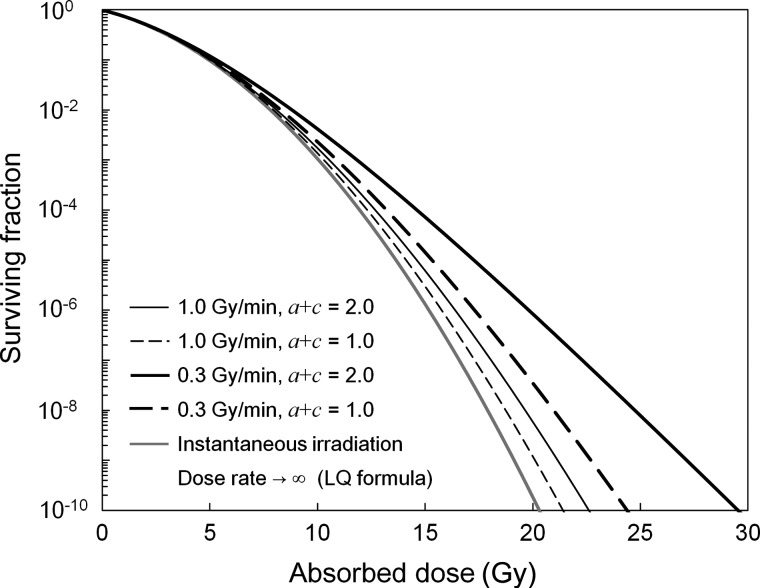
Cell survival curves for a variety of (*a + c*) values in the MK model. The shape has the tendency to become rectilinear above 10 Gy or more. In this figure, we used the MK parameters *α*_0_ and *β*_0_ indicated in Table 1.

To evaluate the nature of SFs described by the MK model visually, the shape of the curves is depicted in Fig. 6 using the parameters, *α*_0_ and *β*_0_ in Table 1, for the combined conditions of the dose rate (1.0 and 0.3 Gy/min) and the value of (*a* + *c*) (2.0 for an exponential phase with tumor cells and 1.0 for cells in the plateau phase [20]). In addition to this, SF in a case of an extremely high dose rate is plotted in Fig. 6, which corresponds to a LQ curve. As in Fig. 6, the difference of the curves begins at around the 6–10 Gy range. This boundary value is in accordance with the report by Park *et al*. that the threshold dose (*D_T_*) to shift from the LQ curve to a straight line in the universal survival curve (USC) is 6.2 Gy [38]. Our investigations strongly suggest that the linearity of the cell survival curve in the high dose range is attributable to the kinetics of DNA lesions (i.e. repair processes) during the dose-delivery time. This characteristic of the rectilinear shape depicted by the MK model would have some effects on both evaluation and prediction of biological effects caused by radiation in normal tissue (or organs at risk in radiotherapy).

## CONCLUSION

In this study, we deduced the MK formula of SF for continuous irradiation with a dose-delivery time (*T*). We determined parameters in the MK model from experimental survival curves for a variety of dose rates. The absorbed dose (*D*) delivered was changed according to the dose rate for the constant dose-delivery time (10 min). It was shown that the dose rate effect on the SF can be reproduced by the MK formula, and the tendency towards rectilinearity for the SF at the high dose region is attributable to the dose rate or the irradiation time, which is in turn related to repair processes of lesions occurring during the dose-delivery time. Therefore, when we evaluate the effects of radiation on cells, we cannot ignore the dose-delivery time for a given dose rate.

## FUNDING

Funding to pay the Open Access publication charges for this article was provided by Hiroyuki Date (Faculty of Health Sciences, Hokkaido University).
